# The translucens group of *Xanthomonas translucens*: Complicated and important pathogens causing bacterial leaf streak on cereals

**DOI:** 10.1111/mpp.12909

**Published:** 2020-01-21

**Authors:** Suraj Sapkota, Mohamed Mergoum, Zhaohui Liu

**Affiliations:** ^1^ Institute of Plant Breeding, Genetics, and Genomics University of Georgia Griffin Campus, Griffin GA USA; ^2^ Department of Crop and Soil Sciences University of Georgia Griffin Campus, Griffin GA USA; ^3^ Department of Plant Pathology North Dakota State University Fargo ND USA

**Keywords:** bacterial diseases, host resistance, pathogen virulence, wheat, *Xanthomonas*

## Abstract

**Abstract:**

*Xanthomonas translucens* is a group of gram‐negative bacteria that can cause important diseases in cereal crops and forage grasses. Different pathovars have been defined according to their host ranges, and molecular and biochemical characteristics. Pathovars have been placed into two major groups: translucens and graminis. The translucens group contains the pathovars causing bacterial leaf streak (BLS) on cereal crops such as wheat, barley, triticale, rye, and oat. In recent years, BLS has re‐emerged as a major problem for many wheat‐ and barley‐producing areas worldwide. The biology of the pathogens and the host–pathogen interactions in cereal BLS diseases were poorly understood. However, recent genome sequence data have provided an insight into the bacterial phylogeny and identification and pathogenicity/virulence. Furthermore, identification of sources of resistance to BLS and mapping of the resistance genes have been initiated.

**Taxonomy:**

Kingdom Bacteria; Phylum *Proteobacteria*; Class *Gammaproteobacteria*; Order *Xanthomonadales*; Family *Xanthomonadacea* *e*; Genus *Xanthomonas*; Species *X. translucens*; translucens group pathovars: *undulosa*, *translucens*, *cerealis*, *hordei*, and *secalis*; graminis group pathovars: *arrhenatheri*, *graminis*, *poae*, *phlei*; newly established pathovar: *pistaciae*.

**Host range:**

*X. translucens* mainly infects plant species in the Poaceae with the translucens group on cereal crop species and the graminis group on forage grass species. However, some strains have been isolated from, and are able to infect, ornamental asparagus and pistachio trees. Most pathovars have a narrow host range, while a few can infect a broad range of hosts.

**Genome:**

The complete genome sequence is available for two *X. translucens* pv*. undulosa* strains and one pv. *translucens* strain*.* A draft genome sequence is also available for at least one strain from each pathovar. The *X. translucens* pv*. undulosa* strain Xt4699 was the first to have its complete genome sequenced, which consists of 4,561,137 bp with total GC content approximately at 68% and 3,528 predicted genes.

**Virulence mechanisms:**

Like most xanthomonads, *X. translucens* utilizes a type III secretion system (T3SS) to deliver a suite of T3SS effectors (T3Es) inside plant cells. Transcription activator‐like effectors, a special group of T3Es, have been identified in most of the *X. translucens* genomes, some of which have been implicated in virulence. Genetic factors determining host range virulence have also been identified.

## Graphical abstract

This pathogen profile summarizes the current knowledge on *Xanthomonas translucens* that causes bacterial leaf streak on cereals.

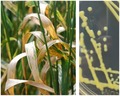

## 1 INTRODUCTION

According to Bamberg (1936), the occurrence of leaf streak‐like disease on wheat and barley had been observed and recorded as early as 1893. However, the formal description of the disease and the causal bacterium was not made until 1917, when Jones and coworkers published the identification of bacterial blight on barley (Jones *et al.*, 1917). The causal bacterium was named *Bacterium translucens* because of the translucent lesions on diseased leaves. Shortly after this the disease on wheat was described, but was named as black chaff, which referred to the disease symptoms on the spikes (Smith *et al.*, 1919). Since then, leaf streak disease has been reported on other small grains and some grasses (Reddy *et al.*, 1924; Hagborg, 1942; Wallin and Reddy, 1945; Fang *et al.*, 1950; Cunfer and Scolari, 1982). On forage grasses, a group of genetically related bacteria cause bacterial wilt (Egli *et al.*, 1975; Egli and Schmidt, 1982).

The classification and nomenclature of bacteria causing bacterial leaf streak (BLS) diseases has been very confusing and has undergone many changes, largely due to their morphological and biochemical similarity and overlapping host range. For a long time, these bacteria were classified as different pathovars (pv.) under the species of *Xanthomonas campestris* (Dye and Lelliott, 1974). Vauterin *et al. *(1992, 1995) proposed the re‐establishment of the species *Xanthomonas* *translucens* including strains that cause leaf streak on small grains and some grasses (the “translucens” group) and strains that cause bacterial wilt on forage grasses (the “graminis” group). This classification and nomenclature system has been supported by recent molecular and whole genome sequence data (Peng *et al.*, 2016; Langlois *et al.*, 2017; Hersemann *et al.*, 2017). Genomic sequence data also suggest that the pathovars cerealis could be genetically separated from other translucens and graminis group pathovars.

Wheat and barley BLS diseases have been found in almost all wheat‐ or barley‐growing areas worldwide (Bamberg, 1936; Duveiller, 1990) and they can cause substantial yield losses and poor grain quality (Waldron, 1929; Shane *et al.*, 1987; Duveiller and Maraite, 1993). Although BLS epidemics have been sporadic and usually occur in warm and humid subtropics regions, in the last decade the incidence of BLS has dramatically increased in the Midwestern United States, where the majority of the hard red spring wheat and durum wheat are produced (Adhikari *et al.*, 2011). Most of the cultivars in this region, as well as other places, are highly susceptible and no chemical methods are available for BLS control in the field (McMullen and Adhikari, 2011). Furthermore, breeding for resistant wheat and barley cultivars is difficult due to the lack of sources of resistance and our knowledge regarding host–pathogen interactions.

Although research has been done on some aspects of the disease system, in particular the recent genome sequencing projects, there has been no review for this group of bacterial pathogens as well as the diseases they cause except a handbook published in 1997 by the International Maize and Wheat Improvement Center (CIMMYT) (Duveiller *et al.*, 1997). In this work, we provide a comprehensive review mainly of the “translucens” group of *X. translucens* pathogens that cause BLS on small grains. This review summarizes the current knowledge regarding the diseases, virulence, and genomics of the bacterial pathogens, and the genetics of host resistance. We also provide some thoughts on the future direction of the research aiming to solve this disease problem.

## 2 DISEASE SYMPTOMS, DISTRIBUTION, AND IMPORTANCE

**Figure 1:**
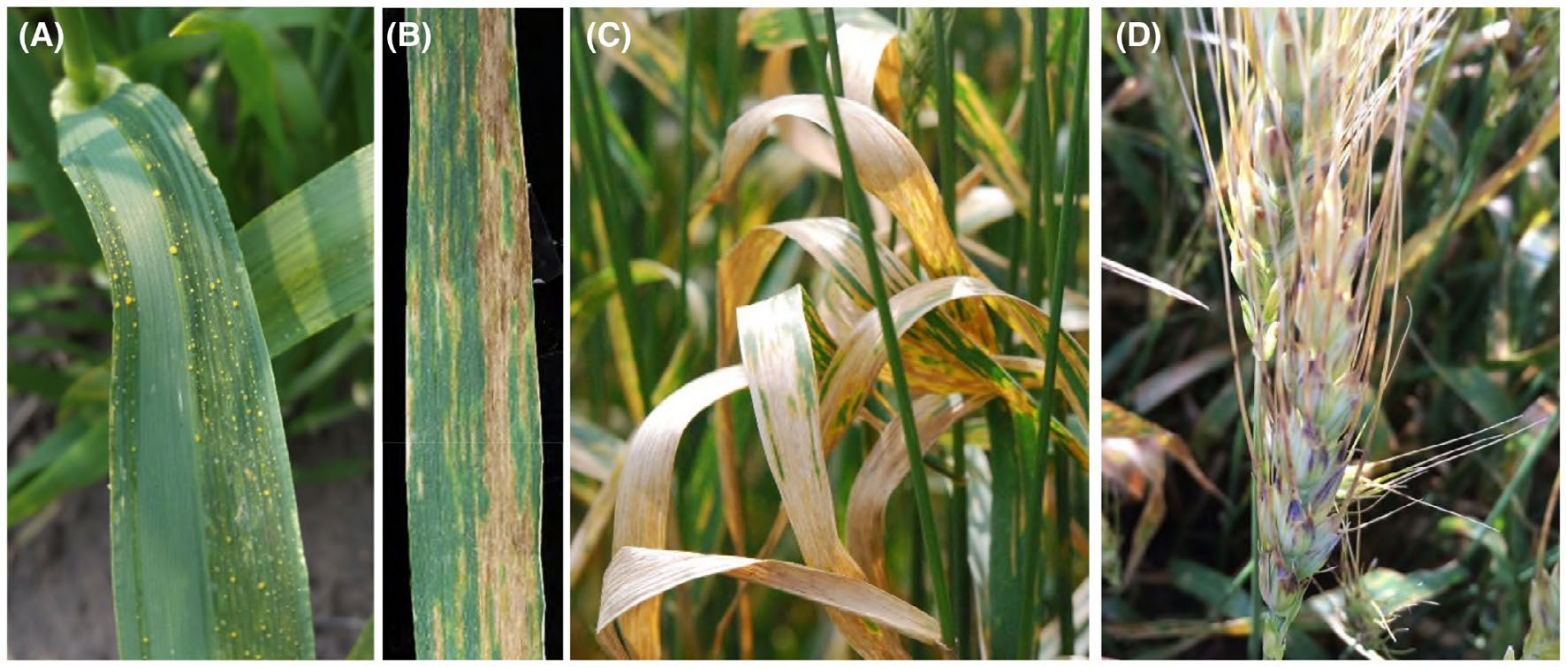
Symptoms and signs associated with wheat bacterial leaf streak. (a) Wheat leaves at the early stage of disease development. Water‐soaking streaks are present with bacterial ooze. (b) Wheat leaf with longitudinal necrotic lesions caused by the bacterium. (c) Completely dead flag leaves caused by the bacterium. (d) Black chaff symptoms on the spike. (Photographs (a) and (d) were kindly provided by Justin Stanton, University of Minnesota, and Dr Erick DeWolf, Kansas State University, respectively)

The symptoms of BLS disease are mainly observed on leaves and spikes. On the leaf, initially water‐soaked streaks develop that subsequently become translucent necrotic lesions (Figure 1a,b). Under warm and humid conditions, bacterial ooze (yellow exudates) can be seen on the leaf surface (Figure 1a). Under high disease pressure, the whole leaf area may be severely affected by the pathogen (Figure 1c). The disease, black chaff, refers to the dark‐purple streaks on the glumes (Figure 1d). The diagnosis of BLS is sometime difficult in the field mainly because the symptoms resemble those caused by fungal pathogens or genetic or environmental factors (Duveiller *et al*., 1997; McMullen and Adhikari, 2011). For example, wheat cultivars that possess stem rust resistance gene *Sr2* develop a melanic reaction that mimics the black chaff symptoms caused by the *X. translucens* pathogens (Duveiller *et al.*, 1993).

BLS disease has been reported from many geographical regions worldwide where wheat is grown. According to Duveiller *et al. *(1997), the occurrence of BLS has been reported from the countries in North America (United States, Canada, Mexico), South America (Argentina, Bolivia, Brazil, Paraguay, Peru, Uruguay), Asia (China, Iran, India, Pakistan, Syria, Turkey, Kazakhstan, Yemen, Israel, Russia, Malaysia, Japan), Africa (Kenya, Ethiopia, South Africa, Tanzania, Tunisia, Libya, Madagascar, Morocco, Zambia), most parts of Europe (France, Romania, Russia, Turkey, Ukraine), and Australia. Recently, the global distribution of BLS has been updated on the web page of the European Plant Protection Organization (EPPO) Global Database (https://gd.eppo.int/taxon/XANTTR/distribution, accessed 20 June 2019). It appears that BLS disease has not been reported from western Europe, which is probably due to unfavourable environmental conditions and extensive quarantine efforts (Paul and Smith, 1989; Duveiller *et al.*, 1997). In the United States, BLS was initially reported in barley and wheat fields in the Midwest (Jones *et al.*, 1917; Smith *et al.*, 1919). Since then, BLS has been found in many places in the United States with outbreaks and epidemics mostly having occurred in the southeastern regions (Milus and Mirlohi, 1994; Tubajika *et al.*, 1999). In recent years, the incidence of BLS has increased in the Upper Midwest region of the United States, including North Dakota, Minnesota, and South Dakota (Adhikari *et al.*, 2012a; Kandel *et al.*, 2012; Curland *et al.*, 2018).

Although BLS is considered as a potential threat for wheat production worldwide, there are no recent reports on yield losses caused by BLS. Furthermore, the importance of BLS varies in different wheat‐growing regions and depends mainly on the level of resistance/susceptibility of wheat cultivars grown and the prevalent environmental conditions. Yield losses due to BLS are generally reported to be 10% or less, but severe infection can cause up to 40% yield losses on highly susceptible cultivars (Waldron, 1929; Forster and Schaad, 1988). Studies have indicated that yield loss due to BLS is generally negatively correlated with BLS severity on flag leaves, and up to 20% yield reduction is possible if 50% leaf area of the flag leaves is infected (Shane *et al.*, 1987; Duveiller and Maraite, 1993). Yield losses are usually due to the reduction in grain weight and the number of kernels per spikes; however, severe infection can cause sterile spikes leading to a complete yield loss (Forster and Schaad, 1988; Tubajika *et al.*, 1998). In addition, BLS infection can alter the protein content of the grains, resulting in quality reduction (Shane *et al.*, 1987).

## 3 DISEASE CYCLE, EPIDEMIOLOGY, AND MANAGEMENT

Many aspects of the BLS aetiology have not been experimentally tested, but a general disease cycle has been proposed (Duveiller *et al.*, 1997, Figure 2). Seed is thought to be an important source of primary inoculum for BLS (Boosalis, 1952; Tsilosani *et al.*, 1977; Timmer *et al.*, 1987; Forster and Schaad, 1988; Milus and Mirlohi, 1995; Rashid *et al.*, 2013). The survival rate of bacteria on seeds and the possibility of transmission to seedlings were shown to be largely dependent on the storage conditions, the length of storage, and the level of susceptibility of genotypes (Boosalis, 1952; Forster and Schaad, 1990; Milus and Mirlohi, 1995). It has been reported that if the number of bacteria is less than 1,000 cfu per gram in the seed lots, no BLS symptoms could be visible in the emerged plants (Klykov, 1945; Duveiller *et al.*, 1997). Studies have shown that weeds and grasses may serve as overwintering hosts or green bridges for the bacterium to spread from one season to other (Wallin, 1946; Fang *et al.*, 1950; Boosalis, 1952; Thompson *et al*., 1989). The bacteria are also able to survive in soil and crop debris for a short period of time (Milus and Mirlohi, 1995; Duveiller *et al.*, 1997; Stromberg *et al.*, 2000).

**Figure 2:**
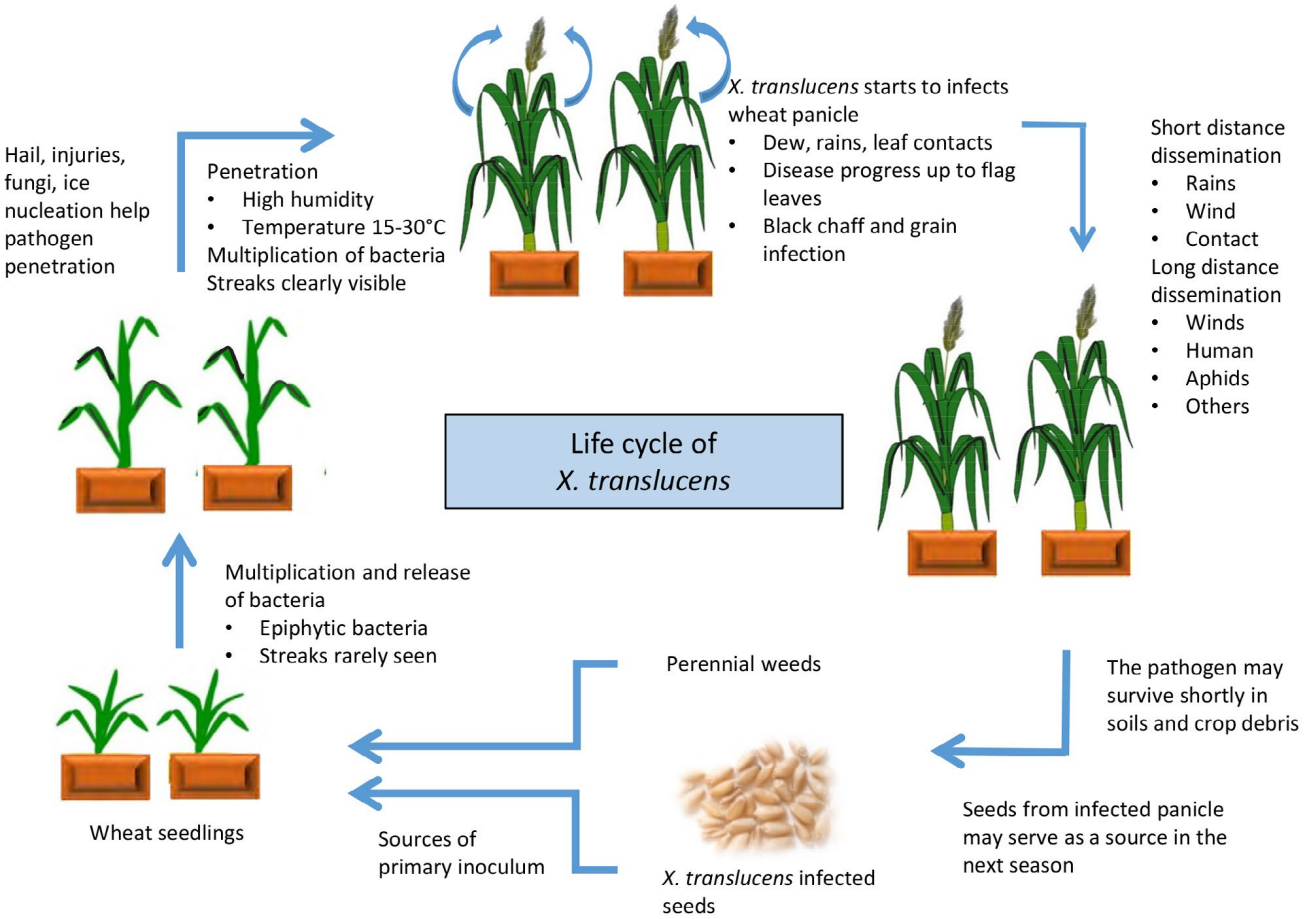
Life cycle of *Xanthomonas translucens* pathogens. The life cycle picture was redrawn based on the descriptions in Duveiller *et al*. (1997)

Although *X. translucens* pathogens enter plant tissues through natural openings, some of them have been reported to possess ice nucleation ability that can cause frost damage leading to the formation of wounding sites for direct entry (Kim *et al.*, 1987; Azad and Schaad, 1988; Gurion‐Sherman and Lindow, 1993). Warm and humid conditions are thought to be important for BLS development because the disease has been found more in wet seasons or in sprinkler‐irrigated fields with warm temperature; however, the exact conditions conducive to BLS development are not well known. BLS epidemics have been reported to be sporadic and vary from year to year (Bamberg, 1936; Duveiller *et al.*, 1991; Tubajika *et al.*, 1998). Artificial introduction of disease in field plots is challenging which makes it difficult to determine the relationship between environmental conditions and BLS epidemics. Duveiller and Maraite (1995) showed that temperature is more important than other factors to initiate epidemics. Other factors implicated in BLS epidemics include damage from wind, hail and frost, dew period, and host genotypes (Duveiller *et al.*, 1997).

Several cultural practices may help reduce BLS incidence; however, limited information is available on their efficacy (Duveiller *et al.*, 1997; Adhikari *et al.*, 2011). Crop rotation is not considered as a major control strategy because the bacteria cannot survive in debris for a long time (Milus and Mirlohi, 1995; Duveiller *et al.*, 1997). Seed is considered an important source of primary inoculum, thus use of clean seed might be a way of reducing BLS incidence. Different methods have been developed to detect the bacterium in seeds, including dilution plating with the use of selective media, seedling infection assays, serodiagnostic assays, PCR amplification, and loop‐mediated isothermal amplification (Forster and Schaad, 1985; Bragard and Verhoyen, 1993; Maes *et al.*, 1996; Langlois *et al.*, 2017). Seed disinfection methods have also been developed to eliminate the bacterium by using chemical or physical methods (Atanasoff and Johnson, 1920; Forster *et al.*, 1990). However, the effectiveness of seed treatment is still questionable because of contradictory results obtained from different studies (Braun, 1920; Duveiller *et al.*, 1997). Using clean seeds or applying seed treatments may reduce disease incidence, but does not stop the spread of BLS inoculum between fields (Duveiller *et al.*, 1997). Very limited studies have been conducted to test chemicals for controlling cereal BLS in the field. Silva *et al. *(2010) tested silicon compounds on BLS disease development but did not obtain conclusive results. Some copper‐based bactericides or antibiotics have been recommended for controlling bacterial diseases caused by other *Xanthomonas* spp. (McManus *et al.*, 2002; Lamichhane *et al.*, 2018), but their effects on cereal *Xanthomonas* spp. have not been tested.

## 4 CLASSIFICATION, NOMENCLATURE, AND IDENTIFICATION OF *X. TRANSLUCENS*

The early classification and nomenclature for this group of bacteria were mainly based on pathogenicity tests and host range. The early taxonomy for *X. translucens* had been very confusing because strains varied greatly in host range and levels of host specificity. Jones *et al. *(1917) first reported BLS disease on barley and named the pathogen *B. translucens*. Later, BLS was reported on wheat by Smith *et al. *(1919) where the pathogen was named *B. translucens* var*. undulosum* because it morphologically resembled the barley pathogen and was able to infect barley through artificial inoculation. Dowson (1939) created the genus *Xanthomonas* to include the species *X*. *translucens* that caused cereal BLS diseases. Based on the natural host as well as the ability to infect hosts using artificial inoculation, Hagborg (1942) classified *X. translucens* into five formae speciales (f. spp.): f. sp. *hordei* (barley), f. sp. *undulosa* (wheat, barley, and rye), f. sp. *secalis* (rye), f. sp. *hordei‐avenae* (barley and oat), and f. sp. *cerealis* (wheat, barley, rye, and oat). Fang *et al. *(1950) argued that f. sp. *cerealis* and *hordei‐avenae* should be combined with f. sp. *undulosa* and *hordei*, respectively, and given the name of f. sp. *cerealis* for strains that naturally occur on smooth bromegrass and quack grass but can infect wheat, barley, rye, and oat using artificial inoculations. Classification by Fang *et al. *(1950) also included f. sp. *phleipratensis*, which was originally identified by Wallin and Reddy (1945) from timothy grass. Because xanthomonad pathogens cannot be easily differentiated by morphology and bacteriological tests, later taxonomic efforts placed the entire *X*. *translucens* f. sp. into *X*. *campestris* as different pathovars, which included *X*. *campestris* pvs. *cerealis*, *hordei*, *secalis*, *translucens*, and *undulosa* (Dye and Lelliott, 1974; Young *et al.*, 1978; Dye *et al.*, 1980).

During the 1970s and 1980s, bacterial wilt was reported on many forage grasses and the causal bacteria were shown to be closely related to *X*. *campestris* identified from cereals (Egli *et al.*, 1975; Wilkins and Exley, 1977; Roberts *et al.*, 1981; Egli and Schmidt, 1982; Channon and Hisset, 1984). Those bacteria were named as different pathovars under *X*. *campestris*, including pv. *graminis,* pv. *arrhenatheri*, pv. *phlei*, and pv. *poae* (Egli and Schmidt, 1982; Van den Mooter *et al.*, 1987). Van den Mooter *et al. *(1987) also recognized that pv. *phlei* is the synonym of pv. *phleipratensis*, which was previously identified by Wallin and Reddy (1945) and Fang *et al. *(1950).

Since the late 1980s, modern DNA, protein, and other biochemical methods have been used in the taxonomy and identification of xanthomonads isolated from cereals and grasses (van den Mooter 1987; Azad and Schaad, 1988; Stead, 1989; Kersters *et al.*, 1989; Vauterin *et al.*, 1992; Rademaker *et al.*, 2006). The main finding was that xanthomonads from cereals and grasses are phylogenetically related, while they can be easily separated by using high‐resolution fingerprinting techniques. Thus, the idea emerged to separate them into two main groups, the “translucens” and “graminis” groups, to describe xanthomonads from cereals and grasses, respectively (Vauterin *et al.*, 1992). Therefore, the subsequent reclassification of xanthomonads made by Vauterin *et al. *(1995) re‐established the species name *X*. *translucens* to encompass all *X. campestris* pathovars infecting cereals (translucens group: pv. *undulosa*, pv. *translucens*, pv. *cerealis*, pv. *hordei*, pv. *secalis*) and grasses (graminis group: pv. *graminis*, pv. *arrhenatheri*, pv. *phlei*, pv. *poae*).

Beside cereals and forage grasses, *X*. *translucens* was also found to infect ornamental asparagus and pistachio trees, which are in the genetically distant plant families Liliaceae and Anacardiaceae, respectively (Rademaker *et al.*, 2006; Marefat *et al*., 2006a, 2006b). Very surprisingly, the bacterial pathogens on asparagus were identified as pv. *undulosa* and were able to infect wheat by cross‐inoculation and vice versa (Rademaker *et al.*, 2006). The bacterium on pistachio trees was designated a new pathovar, *X*. *translucens* pv. *pistaciae,* because it could not be classified into any of pathovars in *X*. *translucens* even though they are closely related (Giblot‐Ducray *et al.*, 2009). The *X*. *translucens* pv. *pistaciae* has been added to the International Congress of Plant Pathology (ICPP) list of plant pathogenic bacteria along with other *X*. *translucens* pathovars (Bull *et al.*, 2010, 2012).

**Figure 3:**
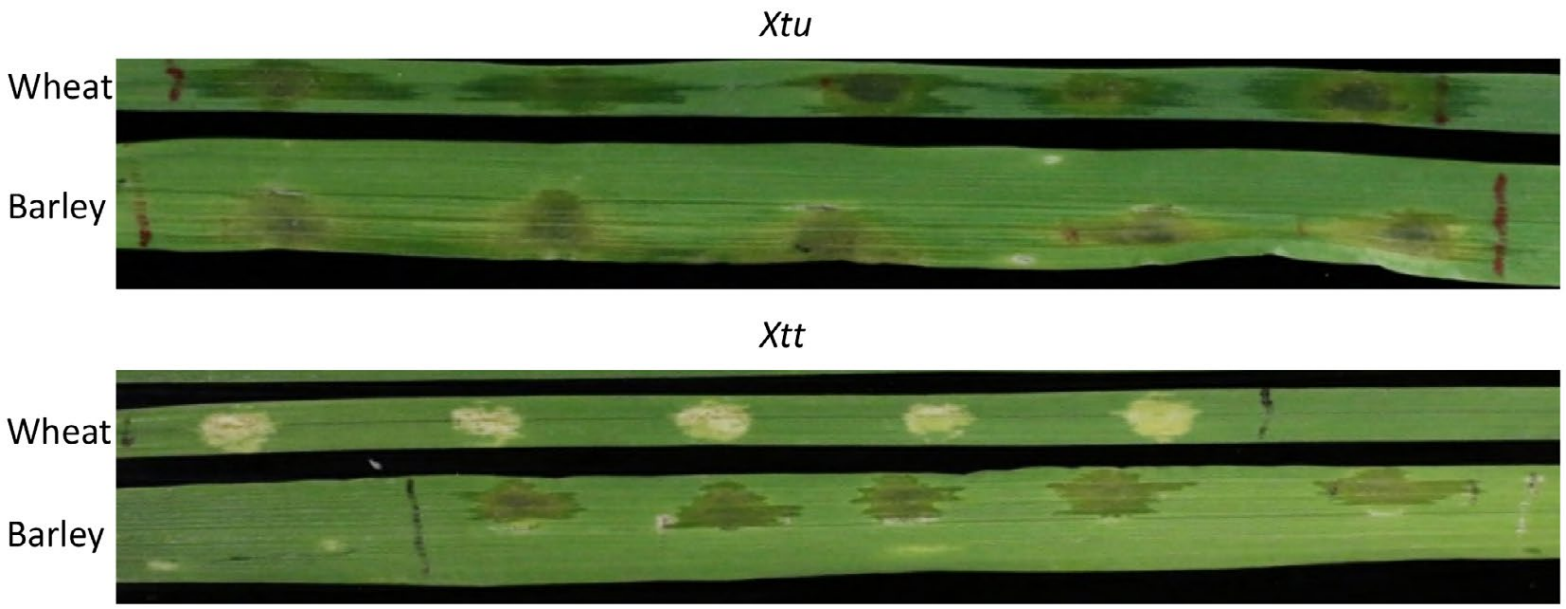
Reaction of wheat or barley to *Xanthomonas translucens* pv. *undulosa* (Xtu) and *X*. *translucens* pv. *translucens* (Xtt) strains. The strains were collected from North Dakota, USA

Further studies have been conducted to examine the genetic similarity and diversity of the pathovars in translucens group by using DNA markers, biochemical profiling, and pathogenicity tests. The studies conducted by Bragard *et al. *(1995, 1997) suggested that *X. translucens* pv. *translucens* is a synonym of *X*. *translucens* pv. *hordei*, and the translucens group contains three true biological entities, *cerealis*, *translucens* and *undulosa*, with pv. *translucens* pathogenic on barley, pv. *undulosa* pathogenic to both barley and wheat, and pv. *cerealis* pathogenic to barley, wheat, oat, and bromegrass. Not many *X. translucens* pv. *secalis* strains were available for analyses, but they were shown to be clustered with pv. *undulosa* (Bragard *et al.*, 1997; Giblot‐Ducray *et al.*, 2009; Curland *et al.*, 2018). Using a few strains isolated from wheat and barley, we showed that wheat strains (*undulosa*) were capable of causing lesions and water‐soaking symptoms on wheat, barley, and triticale (×*Triticosecale*), while barley strains (*translucens*) were only able to cause lesions and water‐soaking symptom on barley (Sapkota *et al.*, 2018; Figure 3). However, Curland *et al. *(2018) reported that most *X. translucens* pv. *undulosa* and pv. *translucens* strains collected from the Upper Midwestern United States cause disease symptoms on both wheat and barley by infiltration, even though their virulence was generally correlated with their original host.

In recent years, *X. translucens* taxonomy has been performed using multilocus sequence analysis with data from the housekeeping gene and/or genome sequences (Wichmann *et al.*, 2013; Gardiner *et al.*, 2014; Peng *et al.*, 2016; Langosis et al. 2017; Curland *et al.*, 2018). The analyses with the DNA sequence data basically agreed with the previous classifications for *X*. *translucens* at both species and pathovar levels determined by DNA–DNA hybridization and DNA marker analysis and pathogenicity tests (Bragard *et al.*, 1997). Evidence has also suggested that cerealis strains could be genetically separated from other translucens and graminis pathovars (Langlois *et al.*, 2017). However, more cerealis strains need to be analysed in order to draw a solid conclusion.

## 5 VIRULENCE and PATHOGENICITY OF *X. TRANSLUCENS*

**Figure 4:**
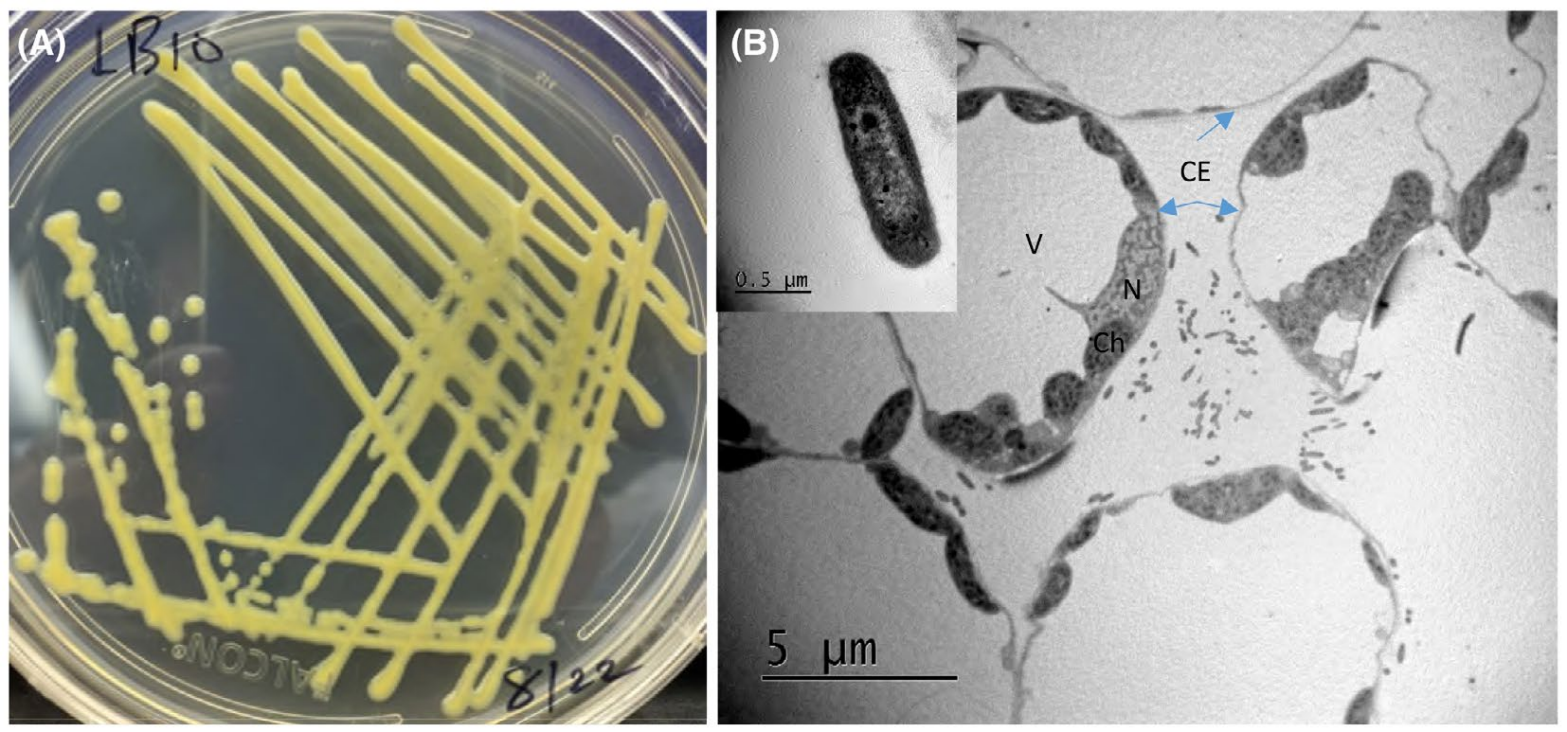
The bacterial pathogen in culture and in planta. (a) Bacterial culture on King's medium B. (b) Scanning electron microscopic photogram of leaf section infected with a *Xanthomonas translucens* pv. *undulosa* strain. The inserted box indicates a single bacterial cell. The parts of the plant cells are indicated: CE, cell envelope (blue arrows); Ch, chloroplast; N, nucleus; V, vacuole

Similar to other xanthomonads, *X*. *translucens* bacteria are gram‐negative, rod‐shaped, non‐spore forming, 0.5–0.8 × 1.0–2.5 µm in size, contain a single polar flagellum, and form pale yellow colonies on nutrient agar medium (Figure 4a,b). Using a transmission electron microscope and the diseased wheat leaf samples collected at the fifth day after spray inoculation with a *X*. *translucens* pv. *undulosa* strain, we observed that bacterial cells were mainly distributed in the mesophyll tissue (Figure 4b). Thus, the bacterial pathogen probably enters plant tissue through stomata and mainly colonizes mesophyll tissues. However, using the same spray inoculation method, we observed little or no disease on barley leaves for *X*. *translucens* pv. *translucens*, suggesting that it may have a different tissue specificity (Liu et al. unpublished data). Leaf clipping and the dip inoculation method have been successfully used in the study by Pesce *et al. *(2017) to induce disease on barley. This suggests that *X*. *translucens* pv. *translucens* probably resides in the vascular tissue. Tissue specificity differences have been reported for other *Xanthomonas* species (Bogdanove *et al.*, 2011). For example, *X*. *oryzae* pv. *oryzae* is a vascular bacterial pathogen causing rice leaf blight, while *X*. *oryzae* pv. *oryzicola*, the cause of bacterial leaf streak, is a nonvascular bacterial pathogen (Bogdanove *et al.*, 2011).

Research in the last few decades has substantially advanced our understanding of the molecular mechanisms of pathogenicity and virulence for *X*. *translucens*. As mentioned above, *X*. *translucens* strains differ in their levels of host specificity, with some capable of infecting multiple host species (broad host range) but others only infecting one host species (narrow host range). The broad host range bacteria may harbour factors controlling virulence rather than narrow‐host range bacteria carrying avirulence factors (Mellano and Cooksey, 1988). Mutants with a narrow host range have been obtained from a wide host range in *X*. *translucens* by using Tn*5* insertion mutagenesis, and host‐specific virulence (*Hsv*) genes have been identified that can restore the ability of the mutants to infect specific hosts (Mellano and Cooksey, 1988; Waney *et al.*, 1991). However, the sequences for these *Hsv* genes have not been determined.

Virulence differences were also observed among strains within a pathovar (Cunfer and Scolari, 1982; Milus and Chalkly, 1994; Adhikari *et al.*, 2011; Sapkota *et al.*, 2018). Using pathogenicity tests on several wheat genotypes, Adhikari *et al. *(2011) found that *X*. *translucens* pv. *undulosa* strains collected from North Dakota are relatively diverse in virulence and there is a significant interaction of wheat–*X*. *translucens* pv. *undulosa*. Previous studies coupled with our recent studies showed that several triticale lines harbour dominant resistance genes to some *X*. *translucens* pv. *undulosa* strains, suggesting the presence of a gene‐for‐gene interaction in triticale (Johnson *et al.*, 1987; Wen *et al.*, 2018). However, it remains unknown if the wheat–*X*. *translucens* pathosystem involves a gene‐for‐gene interaction.

Many gram‐negative bacterial pathogens rely on the type III secretion system (T3SS) and T3SS‐delivered effectors (T3Es) for their pathogenicity and virulence. Phylogenetic analysis showed xanthomonads can be classified into two clades with the majority of species in clade 2, and they usually contain a highly conserved *hrp* gene cluster that encodes the T3SS (Bȕttner and Bonas, 2010; Bogdanove *et al.*, 2011; Pesce *et al.*, 2017). *X*. *translucens* phylogenetically belongs to clade 1, which has four other species: *X*. *albilineans*, *X*. *hyacinthi*, *X*. *theicola*, and *X*. *sacchari* (Young *et al.*, 2008; Gardiner *et al.*, 2014). Among the clade 1 species, *X*. *albilineans* and *X*. *sacchari* have no *hrp* cluster, but all the sequenced *X*. *translucens* strains have it (Pesce *et al.*, 2017). The genetic structure and organization of the *hrp* locus in *X. translucens* genomes is highly conserved among sequenced strains and it is similar to that in clade 1 *Xanthomonas* species and β‐protebacteria (Wichmann *et al.*, 2013; Pesce *et al.*, 2017). Functional analyses have shown that *X. translucens* T3SS is essential for pathogenicity, hypersensitive response induction and effector delivery (Waney *et al.*, 1991; Gardiner *et al.*, 2014; Peng *et al*, 2016; Pesce *et al.*, 2017). However, the *hrp* system in *X*. *graminis* strain Xtg29 has been shown not to be required for pathogenicity but to modulate virulence (Wichmann *et al.*, 2013). This indicates that strains of graminis and translucens may have different biology and virulence mechanisms (Hersemann *et al.*, 2017).

Like other *Xanthamonas* spp., genome sequence data indicate that *X*. *translucens* strains harbour a suite of T3Es (Whichmann *et al*., 2013; Gardiner *et al.*, 2014; Pesce *et al.*, 2015; Peng *et al.*, 2016; Charkhabi *et al.*, 2017; Hersemann *et al.*, 2017). Sequence alignment and comparison showed that a core set of T3Es identified from other *Xanthomonas* spp. was also present in the sequenced *X*. *translucens* genomes. However, variations in the presence/absence and copy number of individual T3Es were detected among different pathovars and among different strains within a pathovar, and also frame‐shift and loss‐of‐function mutations of certain T3Es occur among different strains (Wichmann *et al.*, 2013; Peng *et al.*, 2016; Charkhabi *et al.*, 2017). The unique repertoire of T3Es in different strains and pathovars may reflect the adaptation of strains to various hosts or virulence to specific genotypes of a host (Jacques *et al.*, 2016). However, the function of the majority of T3Es in *X*. *translucens* in virulence or colonization have not been tested and determined.

Transcription activator‐like effectors (TALEs) are a special type of T3Es that are only present in *Xanthomonas* and some strains of *Ralstonia solanacearum* (Bogdanove *et al.*, 2010). TALE genes have been identified from three *X*. *translucens* strains with a complete genome sequence as well as a few strains with a draft genome sequence (Pesce *et al.*, 2015; Jaenicke *et al*., 2016; Peng *et al.*, 2016; Charkhabi *et al.*, 2017). *X*. *translucens* TALEs that have been well characterized were from two *X*. *translucens* pv. *undulosa* strains, Xt4699 and ICMP11055, which have eight and seven genes, respectively. Based on the sequences of repeat variable di‐residue (RVD) amino acids, it was found that four TALEs are common between the two strains and the rest are either partially conserved or have no obvious similarity (Charkhabi *et al.*, 2017). Sequence comparison revealed that the two strains had several unique RVDs, including KG, QD, Y*, YK and YD, which have not been identified from other *Xanthomas* spp. (Peng *et al.*, 2016; Charkhabi *et al.*, 2017). It was also found that some TALEs that are widely distributed in other xanthomonads are less frequently present in *X*. *translucens* (Charkhabi *et al.*, 2017).

TALEs function to induce host gene expression after binding to the promoter region of a gene. Using microarray analysis and TALE gene mutants, Peng *et al. *(2016) provided evidence that TALEs in XT4699 induce high expression of specific wheat genes. For example, the Tal6 was shown to particularly up‐regulate two wheat genes, *Ta.7291.1.S1_S1_at* (a succinate dehydrogenase subunit) and *Ta.14164.1S1_x_at* (bHLH family transcription factor). Very recently, it was found that Tal8 is associated with enhanced virulence in XT4699 by modulating ABA biosynthesis and production (Peng *et al.*, 2019). Two TALE genes in ICMP11055 were also shown by site‐directed mutagensis to contribute to bacterial virulence based on lesion length (Charkhabi *et al.*, 2017). More work is needed to investigate the roles of TALEs in virulence and their underlying molecular mechanisms.

## 6 GENOMICS of *X. TRANSLUCENS*

As mentioned above, genome sequencing and comparative genomics is a powerful tool to reveal candidate genes’ underlying virulence/pathogenicity as well as bacterial classification of the bacterial pathogens, and whole‐genome sequencing has been performed for *X*. *translucens*. The first genome sequence of *X. translucens* was obtained from *X*. *translucens* pv. *graminis* strain Xtg29 (Wichmann *et al.*, 2013). So far, a total of 51 *X*. *translucens* genome assemblies have been deposited in the NCBI Genome Resources (https://www.ncbi.nlm.nih.gov/genome/genomes/14066, accessed 20 June 2019), which covers all pathovars in both translucens and graminis groups. Among them, three were sequenced with long‐reads sequencing techniques and had a complete circular genome sequence, including two *X*. *translucens* pv. *undulosa* strains (Xt4699 from the United States, ICMP11055 from Iran) and one *X*. *translucens* pv. *translucens* strain (DSM 18974 from the United States) (Jaenicke *et al*., 2016; Peng *et al.*, 2016; Charkhabi *et al.*, 2017). The main features for the three complete genomes are listed in Table 1. The remaining genome sequences were fragmented containing variable numbers of scaffolds or contigs (see the above web link) and the genome size of these strains ranged from 4.1 to 4.8 Mb. Size difference was observed among different pathovars and also among different strains within a pathovar (Charkhabi *et al.*, 2017; Hersemann *et al.*, 2017). The annotated protein‐encoding genes in published *X. translucens* genomes ranged from 3,160 to 4,413, with pv. *graminis* relatively having fewer. The available genome sequence data and comparative genomics will greatly facilitate our progress in the understanding host, cultivar, and tissue‐specificity in *X*. *translucens* species.

**Table 1:** Summary of *Xanthomonas translucens* pv. *translucens* or pv. *undulosa* strains that have fully sequenced genomes

	*X. translucens* strain		
	ICMP11055	XT4699	DSM 18974^T^
Pathovar name	*undulosa*	*undulosa*	*translucens*
Host of origin	Wheat	Wheat	Barley
Place of collection	Kerman, Iran	Kansas, United States	Minnesota, United States
Genome size (bp)	4,761,583	4,561,137	4,715,357
GC content (%)	67.8	68.1	67.7
Protein coding genes	3,953	3,528	3,736
Ribosomal RNA operons	2	2	2
Transfer RNAs	54	54	54
CRISPR array	1	Not detected	1
TALE genes	7	8	8
Non‐TALE T3E genes	32	32	–
Insertion sequence elements (complete/partial)	83/58	74/56	–
Reference	Charkhabi *et al.*, 2017	Peng *et al.*, 2016	Jaenicke *et al*., 2016

Note

TALE, transcription activator‐like effector; T3E, type III secretion effector; CRISPR, clustered regularly interspaced short palindromic repeats; RNA, ribonucleic acid.

## 7 GENETICS AND MAPPING OF HOST RESISTANCE

Because no chemical control is available, host resistance appears to be the only way to control wheat BLS. Several sources of BLS resistance have been identified in diverse wheat or barley germplasm and related species (Hagborg, 1974; Akhtar and Aslam, 1986; Duveiller *et al.*, 1993; Alizadeh *et al.*, 1994; El Attari *et al.*, 1996; Milus *et al.*, 1996; Tillman *et al.*, 1996; Adhikari *et al.*, 2011; Kandel *et al.*, 2012; Sapkota *et al.*, 2018). These studies revealed a low percentage of resistant lines and no immune or highly resistant materials were found in the wheat germplasm. Nevertheless, several triticale lines were reported to possess high levels of BLS resistance (Cunfer and Scolari, 1982; Johnson *et al.*, 1987; Sapkota *et al.*, 2018).

Using partial and high levels of BLS resistance in small grain crops, the heritability and genetics of BLS resistance were investigated (Duveiller *et al.*, 1993; El Attari *et al.*, 1996; Tillman and Harrison, 1996; Adhikari *et al.*, 2012b; Kandel *et al.*, 2015; Wen *et al.*, 2018). The heritability of BLS resistance in wheat was reported to vary from low to high depending on the resistant lines used (El Attari *et al.*, 1996; Tillman and Harrison, 1996). Classic genetic analysis showed that BLS resistance could be quantitative or qualitative. Duveiller *et al. *(1993) reported a total of five genes (*Bls1*, *Bls2*, *Bls3*, *Bls4*, and *Bls5*) conferring BLS resistance in three resistant wheat cultivars, with *Bls1* present in all three partially resistant wheat cultivars and having the largest effect. Quantitative inheritance was suggested by disease level distribution based on the reaction of 19 wheat cultivars to 81 *X.* *translucens* pv. *undulosa* strains (Milus and Chalkey, 1994). Using three F_2_ populations derived from Terral 101 (resistant), Coker 9877 (moderately resistant), Pioneer 2548 (susceptible), and Coker 9766 (susceptible), Tillman and Harrison (1996) also detected the presence of multiple genes controlling BLS resistance in these wheat cultivars. However, resistance to BLS in a few triticale lines appears to be qualitative and dominant (Johnson *et al.*, 1987; Wen *et al.*, 2018).

To facilitate the use and transfer of host resistance in breeding programmes, quantitative trait locus (QTL) mapping or genome‐wide association studies (GWAS) have been conducted in recent years to identify genomic regions associated with BLS resistance and linked DNA markers. Using a doubled haploid population and a restriction fragment length polymorphism (RFLP) map, El Attari *et al. *(1998) identified three genomic regions associated with BLS resistance, two on chromosome 3H and one on 7H in barley line Morex. Adhikari *et al. *(2012b) conducted the first GWAS of BLS resistance using a panel of 566 spring wheat landraces and diversity array technology (DArT) markers, which led to the identification of five genomic regions on chromosomes 1A, 4A, 4B, 6B, and 7D associated with BLS resistance. Gurung *et al. *(2014) used the same wheat panel and phenotypic data, but with single nucleotide polymorphism (SNP) markers and identified four QTLs, two of which were on similar genomic regions as reported by Adhikari *et al. *(2012b). Kandel *et al. *(2015) identified two simple sequence repeat (SSR) markers on chromosomes 2A (*Xwmc522*) and 6B (*Xbarc134*) associated with resistance using the identity‐by‐descent mapping method. A total of four QTLs distributed on 2B, 6D, 7A, and 7B were identified by Ayana (2017) using a recombinant inbred lines (RILs) population from the cross of a partially resistant (SD52) and susceptible wheat lines (SD1001) and a genetic map consisting of 1,211 SNP markers. Wen *et al. *(2018) developed two triticale populations derived from a highly resistant genotype (Siskiyou) and two highly susceptible genotypes (UC38 and Villax St. Jose), and mapped a major resistance gene, *Xct1*, on chromosome 5R.

## 8 FUTURE RESEARCH DIRECTIONS

BLS is a common disease of wheat and barley that has the ability to cause a significant reduction in yield and quality. In the last decade, BLS has become an increasingly important problem in all major wheat‐ and barley‐growing areas around the world. Because there is a lack of effective management tools and the understanding of the disease system is limited, the impact of BLS disease on the world’s wheat and barley production will continue to rise. It is imperative to have a global research initiative aiming to solve or mitigate the BLS disease problem. We believe that future research should focus on, but not be limited to, the following areas.

First, an immediate need would be to identify chemicals that can be used at the field scale for disease control while waiting for resistant cultivars to be developed. There are a number of copper‐based commercial bactericides that have been recommended for other foliar diseases caused by xanthomonads. These products can be evaluated directly in the field for their efficacy and profitability. At the same time, new types of chemicals and novel delivery systems should be sought for the development of more efficient and profitable management methods.

Second, it is common knowledge that disease control using genetic resistance is the most desirable method as it is sustainable and environmentally friendly. Therefore, breeding programmes should place more emphasis on using current resistant materials and continue to search for novel sources of resistance. Research is needed to investigate the genetics of resistance in the identified wheat cultivars and breeding lines, and to identify DNA markers linked to the resistance genes or QTLs, which help quickly incorporate partial resistance into local cultivars. Although it could take a long time to move the resistance genes from triticale to wheat cultivars, it is worth doing because these genes confer high levels of resistance to BLS.

Third, a standardized, quick protocol is needed to evaluate pathogen virulence and host resistance. Previous studies have used different inoculation methods, for example direct spraying, injection, and infiltration, with a corresponding rating scale that makes the results incomparable. These inoculations and scoring methods should be further investigated in order to establish a good evaluation protocol in both the greenhouse and the field. This is in particularly important for breeding programmes.

Fourth, an effective management strategy relies on a good understanding of the biology of the disease cycles. There are many areas that need to be addressed, including (a) survival of the pathogens, (b) sources of inoculum and their importance under field conditions, (c) environmental factors favourable for disease development under field conditions, and (d) efficient and accurate identification and detection of the pathogens. Some of the research work mentioned above has been done for the winter wheat regions, but the results need to be confirmed and compared for spring wheat regions.

Lastly, a better understanding of host–pathogen interactions in *X. translucens–*cereals is needed for the development of wheat or barley cultivars with durable resistance. Genome sequences and comparative genomics can serve as a powerful tool to identify bacterial genes underlying bacterial virulence/avirulence and host range determinants. The host susceptibility genes that either directly or indirectly interact with these bacterial virulence genes can be subsequently identified, and then can be mutated through gene‐editing technologies (Li *et al.*, 2012).

## Data Availability

Data sharing is not applicable to this article as no new data were created or analysed in this study.
